# Risk of pre-term birth as a function of sleep quality and obesity: prospective analysis in a large Prematurity Research Cohort

**DOI:** 10.1093/sleepadvances/zpad043

**Published:** 2023-11-02

**Authors:** Siobhan Sutcliffe, Peinan Zhao, Luisa Klaus Pilz, Megan Oakes, Antonina I Frolova, Erik D Herzog, Sarah K England

**Affiliations:** Division of Public Health Sciences, Department of Surgery; Alvin J. Siteman Cancer Center; and the Department of Obstetrics and Gynecology; School of Medicine, Washington University in St. Louis, St. Louis, MO, USA; Division of Clinical Research, Department of Obstetrics and Gynecology, School of Medicine, Washington University in St. Louis, St. Louis, MO, USA; Department of Anesthesiology and Intensive Care Medicine and the Experimental and Clinical Research Center, Charite Universitatsmedizin Berlin, Berlin, Germany; Corporate member of Freie Universität Berlin and Humboldt Universität zu Berlin, Berlin, Germany; Department of Obstetrics and Gynecology, MemorialCare Miller Children’s and Women’s Hospital, Long Beach, CA, USA; Division of Maternal-Fetal Medicine and Ultrasound, Department of Obstetrics and Gynecology, School of Medicine, Washington University in St. Louis, St. Louis, MO, USA; Department of Biology, Washington University in St. Louis, St. Louis, MO, USA; Department of Obstetrics and Gynecology, Center for Reproductive Health Sciences, School of Medicine, Washington University in St. Louis, St. Louis, MO, USA

**Keywords:** sleep, pregnancy, obesity, cohort study

## Abstract

**Study Objective:**

To investigate whether poor sleep quality is associated with pre-term birth (PTB) risk, overall and independent of sleep apnea and habitual snoring.

**Methods:**

We used longitudinal data from the Washington University Prematurity Research Cohort to investigate the association between poor sleep quality (defined as a Pittsburgh Sleep Quality Index > 5) and PTB, overall and independent of sleep apnea and snoring (defined by the Berlin questionnaire and prior sleep clinic attendance). Associations were investigated for sleep quality early and throughout pregnancy. Stratified analyses were performed by factors previously shown to modify associations between sleep and PTB (race, pre-pregnancy obesity).

**Results:**

Of the 976 eligible participants, 50.1% experienced poor sleep quality early in pregnancy (<20 completed weeks) and 14.2% delivered pre-term (*n* = 50 without and 89 with poor sleep quality). In multivariable-adjusted analyses, poor sleep quality early in pregnancy was associated with increased PTB risk (hazard ratio [HR] = 1.48, 95% confidence interval [CI] = 1.02–2.14). This association persisted after further adjustment for sleep apnea and snoring (HR = 1.50, 95% CI = 1.02–2.20) and in analyses stratified by race. It varied, however, by pre-pregnancy obesity. Among individuals without obesity, no association was observed between poor sleep and PTB (HR = 1.08, 95% CI = 0.65–1.79), whereas among those with obesity, a positive association was observed (HR = 2.94, 95% CI = 1.52–5.69, *p*-interaction = .05). This association was limited to individuals with obesity who experienced poor sleep both earlier and later in pregnancy (HR = 3.94, 95% CI = 1.56–9.99).

**Conclusion:**

Our findings suggest that improving sleep quality early in pregnancy may be important for PTB prevention, particularly among individuals with obesity.

## Introduction

Approximately 10% of all infants are born pre-term, defined as a live birth before 37 completed weeks of gestation [1, 2]. These infants are at increased risk for numerous adverse neonatal outcomes, including respiratory distress, seizures, and neonatal death, as well as numerous adverse long-term outcomes, including neurologic and developmental impairments [3]. Pre-term birth (PTB) also contributes to over $26 billion in healthcare and additional costs in the US per year [4]. Despite this high burden, few modifiable risk factors have been identified for PTB to help guide prevention strategies.

One potential modifiable risk factor for PTB is poor sleep quality, including short sleep duration, difficulties falling and staying asleep, and sleep disturbances [5–8]. In recent meta-analyses, individual contributors to poor sleep quality, such as sleep apnea (odds ratios [ORs] = 1.56–1.92 [9, 10]) and habitual snoring (OR = 2.77 [9]), have been consistently associated with an increased risk of PTB in pregnant individuals. These factors have been proposed to raise PTB risk by several potential mechanisms, including increased inflammation, greater oxidative stress, and endothelial dysfunction [5]. Although additional factors besides sleep apnea and snoring can also contribute to poor sleep quality, such as physical discomfort, nocturia, back pain, and leg cramps [11], fewer studies have examined the relationship between overall poor sleep quality and PTB, and none, to our knowledge, have examined this relationship independent of sleep apnea and snoring. Therefore, we used data from the large Washington University Prematurity Research Cohort to investigate the relationship between poor sleep quality and risk of PTB overall, and independent of sleep apnea and habitual snoring. Notably, this cohort included a large proportion of minoritized and disadvantaged pregnant individuals and collected data at multiple time points during follow-up, allowing us to investigate the association between poor sleep quality and PTB throughout pregnancy among individuals at highest risk of PTB.

## Materials and Methods

We reported study methodology and results following the Strengthening the Reporting of Observational Studies in Epidemiology (STROBE) Statement for cohort studies [12].

### Study population, design, and procedures

The Washington University Prematurity Research Cohort [13] was a prospective, longitudinal cohort of pregnant individuals designed to study risk factors for PTB and other maternal and neonatal outcomes. Participants were recruited by convenience sampling from January 2017 to January 2020 at their first prenatal visit at one of two obstetric clinics on the Washington University Medical Campus. Consented participants were required to: (1) be ≥18 years of age; (2) speak English; (3) not be incarcerated; (4) be pregnant with a singleton gestation; (5) have conceived without the aid of in vitro fertilization; (6) be ≤20 weeks of gestation at enrollment; and (7) deliver at Barnes-Jewish Hospital. Study participation involved attending in-person study visits approximately once per trimester (i.e. first, second, and third trimesters) and completing numerous questionnaires, including the Pittsburgh Sleep Quality Index (PSQI), at each of these three in-person visits. Delivery and neonatal data were collected at the time of delivery from the medical record. This study was approved by the Institutional Review Board at Washington University in St. Louis School of Medicine.

Twelve hundred and sixty pregnant individuals were enrolled in the study and delivered at our study hospital. For analyses of poor sleep quality early in pregnancy, we excluded participants who did not have a live birth (*n* = 18) or complete the PSQI at enrollment (i.e. by 20 completed weeks of gestation, *n* = 266). For analyses of poor sleep quality throughout pregnancy, we further excluded participants who did not complete the PSQI at any visit beyond 20 completed weeks of gestation (i.e. at either their second or third trimester visit; *n* = 142). After these exclusions, 976 participants remained in the analyses of poor sleep quality early in pregnancy and 834 in the analyses of poor sleep quality throughout pregnancy.

### Sleep assessment

Sleep quality was measured by the PSQI [6], a 19-item validated scale designed to measure seven components of sleep quality and disturbance over the past month (subjective sleep quality, sleep latency, sleep duration, habitual sleep efficiency, sleep disturbances, use of sleep medication, and daytime dysfunction). The global PSQI is calculated as the sum of each of the seven components (scales of 0–3), for a total range of 0–21. Sleep apnea and habitual snoring were assessed using the validated Berlin Sleep Questionnaire [14] and self-reported previous attendance at a sleep clinic. The 10-item Berlin questionnaire includes items on snoring, breath-holding, daytime sleepiness, and self-reported high blood pressure. These items are combined into one sleep apnea risk category related to snoring and breath-holding, a second related to daytime sleepiness, and a third related to high blood pressure and obesity; these three categories are then summed to create a score. Because we planned to stratify the analyses by obesity a priori, only the first two categories were used in the analysis (modeled as positive for zero, one, or two risk categories). In sensitivity analyses, we also included terms for frequency of snoring and breath-holding, as well as another variable for sleep apnea derived from the validated Epworth Sleepiness Scale (score > 16) [15]. With the exception of previous attendance at a sleep clinic, all other sleep measures were assessed at each of the three in-person study visits.

### PTB assessment

PTB was defined as birth at <37 completed weeks of gestation. Gestational age was determined using data collected at the prenatal visit (best obstetric estimate including last menstrual period or earliest ultrasound dating available [16]) and delivery. These were obtained from the medical record.

### Covariate assessment

Additional variables considered in the analysis were self-reported sociodemographic, clinical, and other factors. Sociodemographic factors included age at enrollment, race/ethnicity, income, educational attainment, insurance status, and marital status. Clinical factors included parity; history of pre-pregnancy chronic hypertension, body mass index (BMI), and asthma; and cigarette smoking status at the time of the first in-person study visit. Perceived stress and depression (measured by the Perceived Stress Scale [17] and Edinburgh Perinatal/Postnatal Depression Scale [18], respectively) were also considered.

### Statistical analysis

Associations between sleep quality and PTB were estimated by Cox proportional hazards regression. Participants’ time at risk started at the date of their first in-person visit (when the first PSQI was completed) and ended at the date of PTB or 37 completed weeks of gestation (when participants were no longer at risk for PTB), whichever came first. Sleep quality was evaluated as a continuous variable, a binary variable for poor sleep quality (PSQI value > 5 [6]), and as quartiles. Because each of these variables yielded similar inferences, only the results for poor sleep quality and sleep quality quartiles are presented.

Multivariable adjustment was performed by Cox regression in two steps. The first step involved including all potential confounders listed in the Covariate section in the models and examining their impact on the effect estimate for sleep quality. Covariates were explored as both continuous and categorical variables (see Table 1 for categories). Those found to shift the point estimate for sleep quality appreciably were retained in the final multivariable-adjusted models. We used this parsimonious modeling strategy to facilitate model convergence in stratified analyses. The second multivariable adjustment step involved adding terms for sleep apnea and habitual snoring to the models to investigate the effect of sleep quality independent of these two conditions. These analyses were repeated: (1) including multiple alternative measures of sleep apnea and habitual snoring; and (2) excluding participants with evidence of sleep apnea and snoring, again using multiple different measures. The analyses were also repeated for each of the seven PSQI components to determine whether any of these components (dichotomized at a score > 1 [6]) were more strongly related to PTB than others.

**Table 1.** Sociodemographic, maternal, and pregnancy characteristics of participants in the Washington University Prematurity Research Cohort, 2017–2020

	All	Poor sleep quality^1^ before 20 weeks of gestation		
		No	Yes	*p*-value
*n*	976	487	489	**—**
Sociodemographic characteristics				
Age (years, mean (range))	28.2 (18.0–42.0)	28.5 (18.0–42.0)	27.9 (18.0–41.0)	.07
Self-identified race (%)				
Black	52.2	44.1	60.1	<.01
White	43.8	51.5	36.0	
Other	4.1	4.3	3.9	
Education (%)				
High school degree or less	50.0	41.9	58.1	<.01
College degree	16.2	18.7	13.7	
Graduate degree	23.5	31.2	15.7	
Missing	10.3	8.2	12.5	
Marital status (%)				
Single	57.0	49.3	64.6	<.01
Married	40.7	48.9	32.5	
Other	2.4	1.8	2.9	
Employment (%)				
Yes	73.4	78.0	68.7	<.01
No	20.2	16.6	23.7	
Student	3.1	2.9	3.3	
Missing	3.4	2.5	4.3	
Annual income (%)^2^				
Government assistance	7.2	5.1	9.2	<.01
<$25 000	37.8	30.4	45.2	
$25 000–$74 999	23.4	22.0	24.7	
≥$75 000	31.4	42.1	20.7	
Insurance (%)^2^				
Government	32.6	26.9	38.2	<.01
Individual/group health insurance	57.7	65.5	49.9	
Uninsured	9.4	7.2	11.7	
Maternal and pregnancy characteristics				
Gravida (median (range))	2.0 (1–15)	2.0 (1–11)	2.0 (1–15)	<.01
Parity (median (range))	1.0 (0–13)	1.0 (0–10)	1.0 (0–13)	<.01
Pre-pregnancy body mass index category (%)				
Under and normal weight (<25 kg/m^2^)	42.6	46.8	38.4	<.01
Overweight (25–29 kg/m^2^)	22.8	24.2	21.5	
Class 1 obesity (30–34 kg/m^2^)	15.3	14.4	16.2	
Class 2 obesity or higher (≥35 kg/m^2^)	19.3	14.6	23.9	
Current smoker at enrolment (%)	7.7	4.5	10.8	<.01
Perceived stress level at enrolment (%)^2^^,^^3^				
Low (0–13)	56.4	71.5	41.3	<.01
Moderate [14–26]	38.8	27.1	50.5	
High (≥27)	4.3	0.8	7.8	
Likelihood of depression at enrolment (%)^4^				
Unlikely (0–8)	82.4	92.0	72.8	<.01
Possible [9–13]	12.1	6.4	17.8	
Probable (≥14)	5.5	1.6	9.4	
History of (%)				
Asthma	18.1	14.2	22.1	<.01
Chronic hypertension	9.8	8.0	11.7	.06
Diabetes	4.9	4.5	5.3	.57
Sleep apnea and habitual snoring-related information (%)				
Sleep apnea risk categories^5^				
None	43.6	56.5	30.9	
One	41.9	33.9	49.9	<.01
Two	8.4	3.9	12.9	
Missing	6.0	5.8	6.3	
Sleep apnea^6^	8.8	5.1	12.5	<.01
History of sleep clinic attendance	3.4	2.3	4.5	.12

^1^Defined as a Pittsburgh Sleep Quality Index value >5.

^2^Numbers may not sum to 100% because of missing information.

^3^Based on the Perceived Stress Scale.

^4^Based on the Edinburgh Perinatal/Postnatal Depression Scale.

^5^Based on two sleep apnea risk categories from the Berlin Sleep Questionnaire, one related to snoring and breath-holding and the other related to daytime sleepiness.

^6^Based on the Epworth Sleepiness Scale questionnaire (score >16).

To investigate sleep quality early in pregnancy, we focused on sleep quality before 20 completed weeks of gestation for three reasons: (1) all participants were required to provide data by this time; (2) it encompasses the date of most initial prenatal visits in the United States [19]; and (3) it is still sufficiently early in pregnancy to allow for effective PTB interventions (e.g. vaginal progesterone administration following mid-trimester short cervix diagnosis [20]). If participants provided two PSQI values before 20 weeks of gestation (i.e. during the first trimester and early in the second trimester), the average of these values was used in the analyses. Secondary analyses evaluated sleep quality in the first trimester (≤14 completed weeks) among the subset of participants with information in this trimester.

To investigate sleep quality later and throughout pregnancy, we analyzed the data in two different ways among participants who provided at least two PSQI values. First, we investigated sleep as a time-dependent, cumulative-updated average [21] of PSQI values at each of the three in-person study visits. In these analyses, we used the first PSQI value at the first in-person visit, the average of the first and second PSQI values at the second visit, and the average of the first, second, and third PSQI values at the third visit. Second, we examined the change in sleep quality before and after 20 completed weeks of gestation by taking the average of values provided before and after 20 completed weeks of gestation and creating categories of change between these two time points: ≤5 at both time points, increase to >5 points after 20 weeks of gestation, decrease to ≤5 after 20 weeks of gestation, and >5 at both time points. Secondary analyses also explored the change in sleep quality between the first and later trimesters (i.e. the second and third trimester combined).

Finally, to examine whether associations varied by factors previously observed to modify associations between sleep and PTB (i.e. self-identified race [22] and pre-pregnancy obesity [23]), we performed stratified analyses by these factors. Effect modification was evaluated by including interaction terms in the model and testing the statistical significance of these terms by the likelihood ratio test. This approach was also used to investigate the proportional hazards assumption (i.e. interaction with time). Lastly, to begin to explore mechanisms for observed associations, additional analyses excluded participants with pre-eclampsia, a possible mediator of the association between sleep quality and PTB, and considered induced and spontaneous PTB separately. All statistical analyses were performed using SAS version 9.4.

## Results

### Sleep quality early in pregnancy and PTB

Of the 976 participants included in the analysis of sleep quality early in pregnancy, approximately half self-identified as Black (52.2%) and had a high school degree or less (50.0%, Table 1). In addition, approximately half were single (57.0%), received government assistance/earned less than $25 000 per year (45.0%), and had individual or group health insurance (57.7%). Approximately three-quarters of participants were employed (73.4%). The median gravidity was two pregnancies and the median parity was one birth. With respect to comorbid conditions and behaviors related to PTB, approximately half of participants were overweight or had obesity before pregnancy (57.4%) and 42.9% had moderate to high levels of perceived stress. Smaller percentages of participants smoked cigarettes (7.7%), or had possible or probable depression (17.6%), asthma (18.1%), or chronic hypertension (9.8%). A small percentage of participants had symptoms of sleep apnea (8.8%) or had attended a sleep clinic (3.4%). Compared to all potential participants in the Washington University Prematurity Research Cohort, those who completed the PSQI at enrollment (and were thus included in the analytic sample) were slightly older, and slightly more likely to self-identify as White, have a higher education level, be married and employed, have a higher income and individual/group health insurance, and have provided information on perceived stress (Supplementary Table 1). They were also slightly less likely to have depressive symptoms, comorbidities, and missing information on sleep apnea.

With respect to sleep quality, the median PSQI value before 20 weeks of gestation was 5.5 (interquartile range [IQR]: 4–8) and 50.1% of participants were classified as having poor sleep quality (Table 1). Participants with poor sleep quality were slightly younger; and more likely to self-identify as Black; have a lower education level; be single and unemployed; receive government assistance or earn <$25 000 per year; and have government insurance or be uninsured. They were also more likely to have obesity; smoke cigarettes; and have moderate to high perceived stress; possible/probable depression; medical conditions, including asthma and chronic hypertension; and evidence of sleep apnea or sleep disorders.

With respect to PTB, 837 participants (85.8%) delivered at term and 139 (14.2%) delivered pre-term. Twenty of these viable pre-term infants subsequently died, whereas no participants experienced mortality. In unadjusted analyses, poor sleep quality early in pregnancy was associated with an increased risk of PTB (Table 2 and Supplementary Table 2). Associations attenuated, but remained statistically significant, after adjustment for sociodemographic and clinical factors related to PTB. Additional adjustment for measures of sleep apnea risk or habitual snoring did not affect the hazard ratios (HRs) for poor sleep quality and PTB, irrespective of how sleep apnea and snoring were defined. Similar HRs were also observed when we excluded participants with any evidence of sleep apnea or snoring. Considering individual components of sleep quality, results were strongest for short sleep duration and greater daytime dysfunction than for other sleep components, but none of the results were statistically significant after adjustment (Table 3).

**Table 2.** Associations between early gestational sleep quality (up to 20 weeks) and risk of pre-term birth (PTB) in the Washington University Prematurity Research Cohort, 2017–2020

	Poor sleep quality^1^		*p*-value	PSQI quartiles				*p*-trend
	No	Yes		Q1 (<4)	Q2 (4 to <6)	Q3 (6 to <8)	Q4 (≥8)	
All participants								
*n*	487	489		209	284	211	272	
PTB incidence (%)	10.3	18.2	<.01	10.1	10.6	16.6	19.5	<.01
Unadjusted HR (95% CI)	1.00	1.86 (1.31–2.62)	<.01	1.00	1.06 (0.61–1.85)	1.71 (1.00–2.94)	2.06 (1.24–3.41)	<.01
Adjusted HR (95% CI)^2^	1.00	1.48 (1.02–2.14)	.04	1.00	0.96 (0.55–1.69)	1.40 (0.80–2.45)	1.48 (0.86–2.56)	.06
Adjusted HR (95% CI)^2^^,^^3^	1.00	1.50 (1.02–2.20)	004	1.00	1.02 (0.57–1.79)	1.47 (0.84–2.60)	1.56 (0.88–2.75)	006
Participants without pre-pregnancy obesity								
*n*	346	293		155	196	125	163	
PTB incidence (%)	10.4	14.0	.17	10.3	10.7	10.5	16.5	.10
Unadjusted HR (95% CI)	1.00	1.38 (0.88–2.16)	.16	1.00	1.04 (0.54–2.00)	1.01 (0.49–2.10)	1.68 (0.91–3.12)	.09
Adjusted HR (95% CI)^2^	1.00	1.04 (0.64–1.69)	.89	1.00	0.83 (0.43–1.62)	0.77 (0.36–1.64)	1.00 (0.50–2.01)	.92
Adjusted HR (95% CI)^2^^,^^3^	1.00	1.08 (0.65–1.79)	.78	1.00	0.86 (0.44–1.69)	0.81 (0.38–1.75)	1.06 (0.52–2.19)	.80
Participants with pre-pregnancy obesity								
*n*	141	196		54	88	87	108	
PTB incidence (%)	9.9	24.5	<.01	9.3	10.2	25.29	24.07	<.01
Unadjusted HR (95% CI)	1.00	2.65 (1.46–4.81)	<.01	1.00	1.12 (0.37–3.33)	3.01 (1.14–7.94)	2.78 (1.07–7.25)	<.01
Adjusted HR (95% CI)^2^	1.00	2.89 (1.51–5.53)	<.01	1.00	1.15 (0.38–3.45)	3.09 (1.15–8.33)	2.92 (1.07–8.00)	<.01
Adjusted HR (95% CI)^2^^,^^3^	1.00	2.94 (1.52–5.69)	<.01^4^	1.00	1.11 (0.37–3.35)	3.14 (1.16–8.52)	2.87 (1.02–8.06)	<.01^5^

CI, confidence interval; HR, hazard ratio; PSQI, Pittsburgh Sleep Quality Index.

^1^Defined as a PSQI value >5.

^2^Adjusted for maternal income, parity, pre-pregnancy chronic hypertension, and perceived stress.

^3^Additionally adjusted for categories of sleep apnea risk and visiting a sleep clinic.

^4^ *p*-interaction = .05.

^5^ *p*-interaction = .10.

**Table 3.** Associations between individual sleep quality components early in pregnancy (up to 20 weeks) and risk of pre-term birth (PTB) in the Washington University Prematurity Research Cohort, 2017–2020

	Subjective sleep quality	Sleep latency	Sleep duration	Habitual sleep efficiency	Sleep disturbances	Use of sleep medication	Daytime dysfunction
All participants							
Score ≤1 (N/PTB incidence [%])	733/13.0	639/12.7	810/12.5	873/13.4	477/10.9	895/14.0	765/12.7
Score >1 (N/PTB incidence [%])	243/18.1	337/17.2	156/22.4	93/21.4	499/17.4	81/17.3	211/19.9
Unadjusted HR (95% CI)	1.43 (1.00–2.04)	1.38 (0.99–1.95)	1.94 (1.32–2.85)	1.60 (0.99–2.60)	1.65 (1.17–2.32)	1.26 (0.72–2.18)	1.63 (1.14–2.35)
Adjusted HR (95% CI)^1^	1.25 (0.86–1.82)	1.05 (0.74–1.50)	1.38 (0.93–2.07)	1.17 (0.71–1.94)	1.24 (0.86–1.79)	1.26 (0.72–2.22)	1.38 (0.93–2.03)
Adjusted HR (95% CI)^1^^,^^2^	1.26 (0.85–1.88)	1.05 (0.73–1.50)	1.41 (0.94–2.13)	1.15 (0.69–1.92)	1.23 (0.84–1.79)	1.27 (0.73–2.24)	1.43 (0.95–2.16)
Participants without pre-pregnancy obesity							
Score ≤1 (N/PTB incidence [%])	492/12.0	435/10.6	549/11.1	575/11.3	346/9.5	582/12.2	504/11.1
Score >1 (N/PTB incidence [%])	147/12.2	204/15.2	83/16.9	57/17.5	293/15.0	57/10.5	135/15.6
Unadjusted HR (95% CI)	1.03 (0.61–1.74)	1.49 (0.94–2.35)	1.60 (0.89–2.86)	1.66 (0.85–3.23)	1.63 (1.04–2.56)	0.86 (0.37–1.98)	1.44 (0.87–2.37)
Adjusted HR (95% CI)^1^	0.91 (0.52–1.57)	0.98 (0.60–1.61)	1.05 (0.56–1.97)	1.08 (0.53–2.18)	1.16 (0.71–1.89)	0.67 (0.28–1.59)	1.19 (0.69–2.06)
Adjusted HR (95% CI)^1^^,^^2^	1.00 (0.56–1.78)	1.04 (0.63–1.73)	1.04 (0.55–1.97)	1.12 (0.55–2.26)	1.23 (0.74–2.05)	0.68 (0.29–1.62)	1.25 (0.71–2.23)
Participants with pre-pregnancy obesity							
Score ≤1 (N/PTB incidence [%])	241/14.9	204/17.2	261/15.3	298/17.5	131/14.5	313/17.3	261/15.7
Score >1 (N/PTB incidence [%])	96/27.1	133/20.3	73/28.8	36/25.0	206/20.9	24/33.3	76/27.6
Unadjusted HR (95% CI)	1.90 (1.14–3.14)	1.17 (0.71–1.93)	2.06 (1.22–3.50)	1.46 (0.72–2.96)	1.44 (0.84–2.48)	2.07 (0.99–4.36)	1.87 (1.11–3.17)
Adjusted HR (95% CI)^1^	1.85 (1.08–3.19)	1.07 (0.63–1.80)	1.83 (1.05–3.14)	1.34 (0.64–2.77)	1.34 (0.76–2.38)	2.22 (1.04–4.71)	1.68 (0.96–2.93)
Adjusted HR (95% CI)^1^^,^^2^	1.81 (1.03–3.19)	1.05 (0.62–1.80)	1.78 (1.03–3.09)	1.33 (0.64–2.77)	1.29 (0.72–2.31)	2.23 (1.05–4.77)	1.61 (0.90–2.90)
*p*-interaction^1^^,^^2^	.15	.90	.28	.87	.99	.03	.55

CI, confidence interval; HR, hazard ratio.

^1^Adjusted for maternal income, parity, pre-pregnancy chronic hypertension, and perceived stress.

^2^Additionally adjusted for categories of sleep apnea risk and visiting a sleep clinic.

In analyses stratified by self-identified race, no differences were observed in the association between sleep quality and PTB risk between Black and White participants (*p*-interaction = .41). Differences were, however, observed by pre-pregnancy BMI. Among participants with normal and overweight status, no associations were observed between sleep quality and PTB, particularly after adjustment for sociodemographic and clinical factors (fully-adjusted HR and 95% confidence interval [CI] for normal weight: 1.41, 0.75–2.64; and overweight: 1.00, 0.42–2.39). In contrast, among participants with class 1 and class 2 obesity or higher, positive associations were observed (class 1: 2.74, 1.09–6.88; and class 2 or higher: 2.88, 1.14–7.27). Therefore, all further analyses were stratified by obesity, grouping normal and overweight together and class 1 and class 2 obesity or higher together (Table 2 and Supplementary Table 2).

When we examined each of the seven individual PSQI components separately, findings were strongest for poor subjective sleep quality, short sleep duration, use of sleep medications, and possibly daytime dysfunction among participants with obesity, whereas no associations were observed for sleep latency, habitual sleep efficiency, and sleep disturbances (Table 3). Finally, associations for poor sleep quality persisted when participants with pre-eclampsia were excluded from the analyses (fully-adjusted HR and 95% CI: 4.16, 1.53–11.33), and were similar by type of delivery: spontaneous (3.81, 1.27–11.5) and induced (2.21, 1.03–4.74; *p*-heterogeneity = .58) among participants with obesity.

### Sleep quality throughout pregnancy and PTB

To investigate the period of time during pregnancy when sleep quality is most important for PTB risk, we performed several analyses among participants with at least two PSQI values, one before and one after 20 completed weeks of gestation (*n* = 834). Participants who provided at least two PSQI values were slightly older, and slightly more likely to self-identify as White, have a higher education level, be married and employed, have a higher income and individual/group health insurance, and report lower perceived stress and less depressive symptoms than those who provided only one or no PSQI values (Supplementary Table 1).

In the first analysis of sleep quality throughout pregnancy, we investigated sleep as a time-dependent cumulative-updated variable. This analysis yielded similar results as for sleep quality early in pregnancy (Table 4), most likely because of the strong correlation between sleep quality values over time (*r* = 0.60, degrees of freedom = 832, *p* < .0001). In the second analysis, we examined changes in sleep quality during pregnancy and found that positive associations were limited to participants with poor sleep quality both before and after 20 weeks of gestation who also had obesity. Numbers were smaller for participants whose sleep quality improved or worsened during pregnancy, but there was no suggestion of an association with risk of PTB for these groups of participants (Table 5 and Supplementary Table 3). Associations for sustained poor sleep quality persisted when participants with pre-eclampsia were excluded (fully-adjusted HR and 95% CI: 12.8, 1.46–111.8) and were similar by type of delivery: spontaneous (8.16, 1.05–63.2) and induced (2.69, 1.09–6.68; *p*-heterogeneity = .33 in participants with obesity).

**Table 4.** Associations between sleep quality throughout pregnancy^1^ and risk of pre-term birth (PTB) in the Washington University Prematurity Research Cohort, 2017–2020

	Poor sleep quality^2^		*p*-value	PSQI quartiles				*p*-trend
	No	Yes		Q1 (<4)	Q2 (4 to <6)	Q3 (6 to <8)	Q4 (≥8)	
All participants								
Unadjusted HR (95% CI)	1.00	1.96 (1.29–2.98)	<.01	1.00	1.02 (0.55–1.92)	1.61 (0.88–2.95)	2.17 (1.21–3.90)	<.01
Adjusted HR (95% CI)^3^	1.00	1.46 (0.92–2.32)	.11	1.00	0.84 (0.45–1.60)	1.16 (0.60–2.22)	1.33 (0.68–2.58)	.22
Adjusted HR (95% CI)^3^^,^^4^	1.00	1.48 (0.92–2.37)	.11	1.00	0.85 (0.45–1.62)	1.17 (0.60–2.26)	1.34 (0.68–2.65)	.21
Participants without pre-pregnancy obesity								
Unadjusted HR (95% CI)	1.00	1.31 (0.77–2.24)	.32	1.00	0.97 (0.47–2.02)	1.00 (0.46–2.20)	1.45 (0.67–3.13)	.37
Adjusted HR (95% CI)^3^	1.00	0.72 (0.38–1.33)	.29	1.00	0.73 (0.34–1.57)	0.53 (0.23–1.24)	0.60 (0.25–1.48)	.22
Adjusted HR (95% CI)^3^^,^^4^	1.00	0.76 (0.41–1.44)	.40	1.00	0.77 (0.36–1.68)	0.55 (0.23–1.31)	0.64 (0.26–1.59)	.28
Participants with pre-pregnancy obesity								
Unadjusted HR (95% CI)	1.00	3.31 (1.54–7.09)	<.01	1.00	1.19 (0.35–4.08)	3.22 (1.07–9.72)	3.42 (1.17–10.02)	<.01
Adjusted HR (95% CI)^3^	1.00	3.64 (1.60–8.29)	<.01	1.00	1.23 (0.36–4.25)	3.80 (1.18–12.27)	4.11 (1.30–13.00)	<.01
Adjusted HR (95% CI)^3^^,^^4^	1.00	3.56 (1.53–8.30)	<.01^5^	1.00	1.22 (0.35–4.26)	3.76 (1.14–12.46)	3.97 (1.21–12.97)	<.01^6^

CI, confidence interval; HR, hazard ratio; PSQI, Pittsburgh Sleep Quality Index.

^1^Modeled as a cumulative-updated, time-dependent average.

^2^Defined as a PSQI value >5.

^3^Adjusted for maternal income, parity, pre-pregnancy chronic hypertension, and perceived stress.

^4^Additionally adjusted for categories of sleep apnea risk and visiting a sleep clinic.

^5^ *p*-interaction = .02.

^6^ *p*-interaction = .03.

**Table 5.** Associations between changes in sleep quality during pregnancy and risk of pre-term birth (PTB) in the Washington University Prematurity Research Cohort, 2017–2020

	Change in poor sleep quality^1^ before and after 20 weeks of pregnancy			
	Both ≤5	Increase to >5	Decrease to ≤5	Both >5
All participants				
*n*	300	128	108	298
PTB incidence (%)	8.0	10.9	10.2	17.1
Unadjusted HR (95% CI)	1.00	1.40 (0.72–2.70)	1.14 (0.56–2.32)	2.09 (1.29–3.40)
Adjusted HR (95% CI)^2^	1.00	1.16 (0.59–2.30)	0.95 (0.45–1.99)	1.58 (0.92–2.71)
Adjusted HR (95% CI)^2^^,^^3^	1.00	1.21 (0.60–2.41)	0.97 (0.46–2.04)	1.62 (0.92–2.84)
Participants without pre-pregnancy obesity				
*n*	217	95	66	180
PTB incidence (%)	7.8	12.6	9.1	10.6
Unadjusted HR (95% CI)	1.00	1.68 (0.80–3.52)	0.99 (0.39–2.52)	1.20 (0.62–2.31)
Adjusted HR (95% CI)^2^	1.00	1.12 (0.50–2.50)	0.80 (0.29–2.17)	0.66 (0.31–1.40)
Adjusted HR (95% CI)^2^^,^^3^	1.00	1.31 (0.58–2.95)	0.87 (0.32–2.44)	0.77 (0.35–1.67)
Participants with pre-pregnancy obesity				
*n*	83	33	42	118
PTB incidence (%)	8.4	6.1	11.9	27.1
Unadjusted HR (95% CI)	1.00	0.66 (0.14–3.17)	1.26 (0.40–3.96)	3.48 (1.54–7.89)
Adjusted HR (95% CI)^2^	1.00	0.75 (0.15–3.78)	1.29 (0.40–4.20)	4.19 (1.73–10.2)
Adjusted HR (95% CI)^2^^,^^3^	1.00	0.71 (0.14–3.69)	1.31 (0.40–4.32)	3.94 (1.56–9.99)^4^

CI, confidence interval; HR, hazard ratio.

^1^Defined as a Pittsburgh Sleep Quality Index value >5.

^2^Adjusted for maternal income, parity, pre-pregnancy chronic hypertension, and perceived stress.

^3^Additionally adjusted for categories of sleep apnea risk and visiting a sleep clinic.

^4^ *p*-interaction = .03 for the comparison of both >5 to both ≤5.

## Discussion

In this large prospective study of diverse pregnant individuals, we found that sustained poor sleep quality throughout pregnancy was associated with significantly increased PTB risk among individuals with obesity, overall and independent of sleep apnea and habitual snoring.

Together, our findings offer support for future sleep-directed preventive interventions, as well as possible insight into the optimal nature, timing, and target population of these potential interventions. First, our findings suggest that additional sleep interventions besides sleep apnea and snoring management may reduce PTB risk, as adjustment for self-reported sleep apnea/snoring and exclusion of individuals with evidence of sleep apnea/snoring had minimal to no effect on the HRs for sleep quality. These findings were observed: (1) irrespective of how we measured or analyzed sleep apnea/snoring; and (2) independent of several known correlates or risk factors for poor sleep quality and PTB (e.g. perceived stress, depression, and chronic hypertension), lending further support for a direct association between additional contributors to poor sleep quality and PTB, and motivating additional confirmatory studies using objective measures of both sleep apnea and sleep quality.

Second, analyses of individual sleep components suggest that interventions directed at lesser opportunity for sleep (e.g. going to bed late and/or waking up early) rather than lower quality of sleep (e.g. difficulty falling and remaining asleep because of stress, physical discomfort, nocturia, etc.) may be important based on the contrasting positive association for short sleep duration (<6 hours) and null associations for poor sleep latency (length of time spent falling asleep), poor habitual sleep efficiency (percentage of time spent awake in bed), and greater sleep disturbances. Findings for short sleep duration are consistent with several (but not all) previous studies of sleep duration [9, 10, 24–26]; however, no previous studies, to our knowledge, have examined other PSQI components in relation to PTB or explored reasons for low sleep opportunity. Therefore, additional studies are warranted to confirm and extend our findings. These could include studies using the Munich Chronotype questionnaire or actigraphy to identify possible weekly patterns of sleep associated with PTB (as the PSQI tends to capture sleep quality on work days rather than work-free days [27]), or qualitative studies to better understand reasons for low sleep opportunity (e.g. structural barriers).

Third, our analyses of sleep quality throughout pregnancy suggest that sleep-directed interventions initiated up to 20 completed weeks of gestation could still be effective at reducing PTB birth risk, as participants with poor sleep quality early in pregnancy who experienced improved sleep later in pregnancy had no greater risk of PTB than those with good sleep quality throughout pregnancy. Only participants with sustained poor sleep quality throughout pregnancy were at increased risk of PTB in our analysis. Finally, our stratified findings suggest that sleep-directed interventions may be most appropriate for pregnant individuals with obesity, as associations between poor sleep quality and PTB risk were limited to these individuals. Although additional confirmatory studies will be necessary to rule out the role of chance, these findings are biologically plausible because both poor sleep quality (including chronodisruption and circadian misalignment [28]) and obesity [29] are associated with inflammation, which may potentially synergize to increase the risk of early parturition, itself a pro-inflammatory state.

Strengths of our study include its large sample size; diverse study population; rare longitudinal assessment of sleep quality throughout pregnancy; collection of detailed sleep data, including information on sleep disturbance/quality, sleep apnea, and habitual snoring; and collection of information on multiple potential confounding variables. Limitations include its: (1) inability to evaluate possible acute rather than longer-term effects of poor sleep on PTB (e.g. the acute influence of one or two poor night’s sleep on subsequent PTB); (2) inability to evaluate short-term variability in sleep quality (i.e. within the 1-month recall time frame of the PSQI) and PTB; (3) restriction to pregnant individuals who provided PSQI data, which likely excluded individuals with the least ability to participate in longitudinal, clinic-based studies (e.g. those with lower incomes and possible transportation or childcare barriers); and (4) reliance on self-reported data for all sleep variables. Although self-reported sleep-related items have been found to correlate with objective measures of poor sleep quality in non-pregnant [30, 31] and pregnant individuals [32], confirmation with objective measures is warranted.

In conclusion, we observed that participants with sustained poor sleep quality throughout pregnancy, as well as obesity, had a significantly increased risk of PTB. These findings suggest that sleep interventions directed at individuals with poor sleep quality early in pregnancy and those who have obesity may help to reduce their PTB risk. Future studies should confirm these findings and elucidate the underlying mechanisms to inform the optimal nature of sleep-related preventive interventions.

## Supplementary Material

zpad043_suppl_Supplementary_Tables_S1-S3

## Data Availability

The data are not publicly available, as the minimal data set for this study on pregnant participants contains identifying patient-level data that cannot be suitably de-identified or aggregated. Additionally, a subset of participants did not consent for future research in the patient consent form approved by the Institutional Review Board at Washington University in St. Louis. Proposals for access to this data should be directed to christinekramer@wustl.edu, Senior Clinical Research Coordinator in the Division of Clinical Research in the Department of Obstetrics and Gynecology. To gain access, data requestors will need to sign a data access agreement.
