# Supplementary material for: Risk of pre-term birth as a function of sleep quality and obesity: prospective analysis in a large Prematurity Research Cohort
Source: Sleep Adv. 2023 Nov 2;4(1):zpad043. doi: 10.1093/sleepadvances/zpad043 (PMC10642756; doi:10.1093/sleepadvances/zpad043)
Supplement: zpad043_suppl_Supplementary_Tables_S1-S3 [file zpad043_suppl_supplementary_tables_s1-s3.docx]

Appendix Table 1: Sociodemographic, maternal, and pregnancy characteristics of participants in the Washington University Prematurity Research Cohort by degree of completion of the Pittsburgh Sleep Quality Index (PSQI) throughout pregnancy, 2017-2020.

|  |  | Participants who completed at least one PSQI value **before** 20 weeks of gestation | Participants who completed at least one PSQI value **before and after** 20 weeks of gestation |
| --- | --- | --- | --- |
|  | All participants who delivered a live birth |  |  |
| N | 1,242 | 976 | 834 |
| **Sociodemographic characteristics** | | | |
| Age (years, mean (range)) | 27.9 (18-42) | 28.2 (18.0-42.0) | 28.6 (18-42) |
| Self-identified race (%) |  |  |  |
| Black | 56.0 | 52.2 | 47.0 |
| White | 39.9 | 43.8 | 48.3 |
| Other | 4.2 | 4.1 | 4.7 |
| Education (%) |  |  |  |
| High school degree or less | 52.2 | 50.0 | 45.6 |
| College degree | 14.3 | 16.2 | 18.2 |
| Graduate degree | 20.0 | 23.5 | 26.5 |
| Missing | 13.5 | 10.3 | 9.7 |
| Marital status (%) |  |  |  |
| Single | 61.0 | 57.0 | 52.2 |
| Married | 36.7 | 40.7 | 45.7 |
| Other | 2.3 | 2.4 | 2.2 |
| Employment (%) |  |  |  |
| Yes | 70.6 | 73.4 | 75.3 |
| No | 21.9 | 20.2 | 18.8 |
| Student | 3.1 | 3.1 | 2.9 |
| Missing | 4.4 | 3.4 | 3.0 |
| Annual income (%) |  |  |  |
| Government assistance | 7.3 | 7.2 | 6.5 |
| <$25,000 | 37.2 | 37.8 | 34.5 |
| $25,000-$74,999 | 21.1 | 23.4 | 23.1 |
| ≥$75,000 | 25.4 | 31.4 | 35.6 |
| Missing | 9.0 | 0.3 | 0.2 |
| Insurance (%)^2^ |  |  |  |
| Government | 35.3 | 32.6 | 29.1 |
| Individual/group health insurance | 54.0 | 57.7 | 62.8 |
| Uninsured | 10.5 | 9.4 | 7.9 |
| **Maternal and pregnancy characteristics** | | | |
| Gravida (median (range)) | 2.0 (1-15) | 2.0 (1-15) | 2.0 (1-15) |
| Parity (median (range)) | 1.0 (0-13) | 1.0 (0-13) | 1.0 (0-13) |
| Pre-pregnancy body mass index category (%) | |  |  |
| Under and normal weight (<25 kg/m^2^) | 41.7 | 42.6 | 43.8 |
| Overweight (25-29 kg/m^2^) | 23.3 | 22.8 | 23.1 |
| Class 1 obesity (30-34 kg/m^2^) | 14.9 | 15.3 | 14.3 |
| Class 2 obesity or higher (≥35 kg/m^2^) | 20.1 | 19.3 | 18.8 |
| Current smoker at enrollment (%) | 8.1 | 7.7 | 7.7 |
| Perceived stress level at enrollment (%)^3^ |  |  |  |
| Low (0-13) | 50.2 | 56.4 | 58.5 |
| Moderate (14-26) | 38.5 | 38.8 | 37.3 |
| High (≥27) | 4.3 | 4.3 | 3.7 |
| Missing | 6.9 | 0.5 | 0.5 |
| Likelihood of depression at enrollment (%)^4^ |  |  |  |
| Unlikely (0-8) | 81.2 | 82.4 | 84.2 |
| Possible (9-13) | 12.9 | 12.1 | 11.2 |
| Probable (≥14) | 6.0 | 5.5 | 4.7 |
| History of (%): |  |  |  |
| Asthma | 19.6 | 18.1 | 17.9 |
| Chronic hypertension | 11.2 | 9.8 | 9.1 |
| Diabetes | 5.4 | 4.9 | 5.4 |
| Sleep apnea and habitual snoring-related information (%) | |  |  |
| Sleep apnea risk categories^3^ |  | 43.6  41.9  8.4 |  |
| None | 37.4 |  | 44.7 |
| One | 36.2 |  | 41.0 |
| Two | 7.2 |  | 7.9 |
| Missing | 19.3 | 6.0  8.8  3.5 | 6.4 |
| Sleep apnea^4^ | 8.3 |  | 8.3 |
| History of sleep clinic attendance | 3.1 |  | 3.5 |

^1^ Defined as a Pittsburgh Sleep Quality Index value >5.

^2^ Numbers may not sum to 100% because of missing information.

^3^ Based on the Perceived Stress Scale.

^4^ Based on the Edinburgh Perinatal/Postnatal Depression Scale.

^5^ Based on two sleep apnea risk categories from the Berlin Sleep Questionnaire, one related to snoring and breath-holding and the other related to daytime sleepiness.

^6^ Based on the Epworth Sleepiness Scale questionnaire (score >16).

Appendix Table 2: Associations between early gestational sleep quality (first trimester) and risk of pre-term birth (PTB) in the Washington University Prematurity Research Cohort, 2017-2020.

|  | **Poor sleep quality**^1^ | | **P-value** | **PSQI quartiles** | | | | **P-trend** |
| --- | --- | --- | --- | --- | --- | --- | --- | --- |
|  | **No** | **Yes** |  | **Q1 (<4)** | **Q2 (4 to <6)** | **Q3 (6 to <8)** | **Q4 (≥8)** |  |
| ***All participants*** | | | | | | | | |
| N | 366 | 329 |  | 162 | 205 | 131 | 197 |  |
| PTB incidence (%) | 9.8 | 17.0 | <0.01 | 9.9 | 10.2 | 15.3 | 17.8 | 0.01 |
| Unadjusted HR (95% CI) | 1.00 | 1.80 (1.18-2.73) | <0.01 | 1.00 | 1.05 (0.55-2.01) | 1.59 (0.82-3.01) | 1.89 (1.05-3.41) | 0.01 |
| Adjusted HR (95% CI)^2^ | 1.00 | 1.33 (0.84-2.10) | 0.22 | 1.00 | 0.98 (0.51-1.89) | 1.30 (0.66-2.54) | 1.22 (0.64-2.32) | 0.42 |
| Adjusted HR (95% CI)^2,3^ | 1.00 | 1.38 (0.86-2.20) | 0.18 | 1.00 | 1.00 (0.51-1.94) | 1.33 (0.67-2.65) | 1.28 (0.65-2.52) | 0.31 |
| ***Participants without pre-pregnancy obesity*** | | | | | | | | |
| N | 272 | 189 |  | 128 | 145 | 77 | 111 |  |
| PTB incidence (%) | 10.7 | 11.6 | 0.74 | 10.2 | 11.7 | 6.5 | 14.4 | 0.50 |
| Unadjusted HR (95% CI) | 1.00 | 1.10 (0.63-1.91) | 0.74 | 1.00 | 1.19 (0.58-2.44) | 0.63 (0.22-1.76) | 1.48 (0.71-3.08) | 0.48 |
| Adjusted HR (95% CI)^2^ | 1.00 | 0.78 (0.43-1.43) | 0.42 | 1.00 | 1.01 (0.48-2.13) | 0.49 (0.17-1.41) | 0.88 (0.38-2.05) | 0.51 |
| Adjusted HR (95% CI)^2,3^ | 1.00 | 0.84 (0.44-1.61) | 0.60 | 1.00 | 1.00 (0.47-2.14) | 0.50 (0.17-1.50) | 0.95 (0.39-2.29) | 0.66 |
| ***Participants with pre-pregnancy obesity*** | | | | | | | | |
| N | 94 | 140 |  | 34 | 60 | 54 | 86 |  |
| PTB incidence (%) | 7.5 | 24.3 | <0.01 | 8.8 | 6.7 | 27.8 | 22.1 | <0.01 |
| Unadjusted HR (95% CI) | 1.00 | 3.54 (1.57-7.99) | <0.01 | 1.00 | 0.73 (0.16-3.26) | 3.44 (1.00-11.87) | 2.61 (0.77-8.83) | 0.01 |
| Adjusted HR (95% CI)^2^ | 1.00 | 3.38 (1.44-7.93) | <0.01 | 1.00 | 0.81 (0.18-3.67) | 3.92 (1.13-13.67) | 2.29 (0.64-8.14) | 0.05 |
| Adjusted HR (95% CI)^2,3^ | 1.00 | 3.42 (1.43-8.19) | <0.01^4^ | 1.00 | 0.84 (0.18-3.88) | 4.11 (1.14-14.84) | 2.18 (0.57-8.32) | 0.04^5^ |

CI=confidence interval; HR=hazard ratio; PSQI=Pittsburgh Sleep Quality Index.

^1^ Defined as a PSQI value >5.

^2^ Adjusted for maternal income, parity, pre-pregnancy chronic hypertension, and perceived stress.

^3^ Additionally adjusted for categories of sleep apnea risk and visiting a sleep clinic.

^4^ P-interaction=0.01.

^5^ P-interaction=0.08.

Appendix Table 3: Associations between changes in sleep quality during pregnancy and risk of pre-term birth (PTB) in the Washington University Prematurity Research Cohort, 2017-2020.

|  | **Change in poor sleep quality^1^ between the first and later trimesters** | | | |
| --- | --- | --- | --- | --- |
|  | **Both ≤5** | **Increase to >5** | **Decrease to ≤5** | **Both >5** |
| ***All participants*** |  |  |  |  |
| N | 244 | 87 | 79 | 202 |
| PTB incidence (%) | 7.8 | 11.5 | 11.4 | 17.3 |
| Unadjusted HR (95% CI) | 1.00 | 1.52 (0.71-3.27) | 1.29 (0.58-2.85) | 2.19 (1.25-3.83) |
| Adjusted HR (95% CI)^2^ | 1.00 | 1.02 (0.45-2.32) | 1.04 (0.46-2.39) | 1.49 (0.78-2.86) |
| Adjusted HR (95% CI)^2,3^ | 1.00 | 1.03 (0.45-2.35) | 1.01 (0.44-2.33) | 1.53 (0.78-2.99) |
| ***Participants without pre-pregnancy obesity*** | | | | |
| N | 186 | 66 | 43 | 120 |
| PTB incidence (%) | 8.1 | 15.2 | 9.3 | 9.2 |
| Unadjusted HR (95% CI) | 1.00 | 1.99 (0.89-4.43) | 0.93 (0.31-2.83) | 1.00 (0.46-2.18) |
| Adjusted HR (95% CI)^2^ | 1.00 | 1.21 (0.48-3.06) | 0.92 (0.29-2.94) | 0.69 (0.29-1.65) |
| Adjusted HR (95% CI)^2,3^ | 1.00 | 1.36 (0.52-3.52) | 0.98 (0.30-3.18) | 0.67 (0.26-1.69) |
| ***Participants with pre-pregnancy obesity*** | | | | |
| N | 58 | 21 | 36 | 82 |
| PTB incidence (%) | 6.9 | 0.0 | 13.9 | 29.3 |
| Unadjusted HR (95% CI) | 1.00 | ----- | 1.92 (0.52-7.17) | 4.94 (1.71-14.3) |
| Adjusted HR (95% CI)^2^ | 1.00 | ----- | 2.22 (0.56-8.75) | 6.00 (1.83-19.7) |
| Adjusted HR (95% CI)^2,3^ | 1.00 | ----- | 1.78 (0.43-7.35) | 5.22 (1.50-18.1)^4^ |

CI=confidence interval; HR=hazard ratio.

^1^ Defined as a Pittsburgh Sleep Quality Index value >5.

^2^ Adjusted for maternal income, parity, pre-pregnancy chronic hypertension, and perceived stress.

^3^ Additionally adjusted for categories of sleep apnea risk and visiting a sleep clinic.

^4^ P-interaction=0.02 for the comparison of both >5 to both ≤5.
